# Analysis of the Kinetics of Swimming Pool Water Reaction in Analytical Device Reproducing Its Circulation on a Small Scale

**DOI:** 10.3390/s20174820

**Published:** 2020-08-26

**Authors:** Wojciech Kaczmarek, Jarosław Panasiuk, Szymon Borys, Aneta Pobudkowska, Mikołaj Majsterek

**Affiliations:** 1 Faculty of Mechatronics and Aerospace, Military University of Technology, Kaliskiego 2 Street, 00-908 Warsaw, Poland; wojciech.kaczmarek@wat.edu.pl (W.K.); jaroslaw.panasiuk@wat.edu.pl (J.P.); 2 Faculty of Chemistry, Warsaw University of Technology, Noakowskiego 3 Street, 00-664 Warsaw, Poland; pobudka@ch.pw.edu.pl; 3 Virtual Power Plant Sp. z.o.o., Dubois 114/116 Street premises 2.30, 93-465 Łódź, Poland; mikolaj.majsterek@vpplant.pl

**Keywords:** algorithm, analytical device, PLC, testing of water parameters, THM, OHT

## Abstract

The most common cause of diseases in swimming pools is the lack of sanitary control of water quality; water may contain microbiological and chemical contaminants. Among the people most at risk of infection are children, pregnant women, and immunocompromised people. The origin of the problem is a need to develop a system that can predict the formation of chlorine water disinfection by-products, such as trihalomethanes (THMs). THMs are volatile organic compounds from the group of alkyl halides, carcinogenic, mutagenic, teratogenic, and bioaccumulating. Long-term exposure, even to low concentrations of THM in water and air, may result in damage to the liver, kidneys, thyroid gland, or nervous system. This article focuses on analysis of the kinetics of swimming pool water reaction in analytical device reproducing its circulation on a small scale. The designed and constructed analytical device is based on the SIMATIC S7-1200 PLC driver of SIEMENS Company. The HMI KPT panel of SIEMENS Company enables monitoring the process and control individual elements of device. Value of the reaction rate constant of free chlorine decomposition gives us qualitative information about water quality, it is also strictly connected to the kinetics of the reaction. Based on the experiment results, the value of reaction rate constant was determined as a linear change of the natural logarithm of free chlorine concentration over time. The experimental value of activation energy based on the directional coefficient is equal to 76.0 $\left[kJ\times {mol}^{-1}\right]$. These results indicate that changing water temperature does not cause any changes in the reaction rate, while it still affects the value of the reaction rate constant. Using the analytical device, it is possible to constantly monitor the values of reaction rate constant and activation energy, which can be used to develop a new way to assess pool water quality.

## 1. Introduction

The most common cause of diseases in public bathing sites is the lack of sanitary control of water quality in swimming pools and public water reservoirs. Among the people most at risk of infection acquired in the pool are children; pregnant women; and immunocompromised people, including AIDS patients, transplant patients, or those undergoing chemotherapy. These people may develop severe infections with a severe course. Among the microorganisms that cause serious illnesses in humans are, among others, *Escherischia coli, Staphylococcus aureus, Legionella pneumophila, Pseudomonas aeruginosa, Streptococcus spp.*; viruses: *Coxsackie*, echo viruses, and adenoviruses; infectious hepatitis; numerous protozoa; and fungi [1,2,3,4,5].

Owners of swimming pools and bathing areas try to ensure safe conditions through appropriate use of disinfectants. However, due to the high dynamics of changes in factors influencing the state of water (changes in the intensity of traffic in the pool, quality of tap water, temperature, and use of filters), a common practice is the use of disinfectants too intensively [6,7]. Excessive use of disinfectants is as unfavorable to human health as not using them at all. It can also cause numerous diseases, especially skin and respiratory system diseases [8,9,10,11,12,13]. Therefore, in water facilities, there is still a struggle to achieve a water level that meets microbiological requirements and in which disinfectants are at a level that does not pose a threat to human health. Among the by-products of water disinfection resulting from chlorination are [14] the following.

- Chloramines, which are formed as a result of the reaction of chlorine with ammonium nitrogen (e.g., present in sweat or urine) in the water. They cause a characteristic “chlorine smell” in the pool. Carbon filters and water irradiation with ultraviolet UV light in pool water treatment plants remove chloramines from pool water. As a result, the concentration of chloramine in the pool air is minimized [15,16,17]. In humans, chloramines can irritate the conjunctiva of eyes. Red eyes are often the result of a longer time spent in the pool.
- Trihalomethanes, which are a by-product of chlorine disinfection of the pool water. While determining THMs in water and air there are four main compounds: chloroform, bromoform, dibromochloroform, and bromodichloroform, of which the first one is the most popular (part in mixture 71–72%) [18,19,20,21]. Chloroform is an organic compound, which is insoluble in water, volatile, and heavier than air, through which it easily passes from liquid to gaseous phase, and its higher concentrations can be observed just above the water surface, i.e., in the main area of human presence. Its concentration in the air is much higher in indoor pools than in outdoor pools [22].

The problem of water quality is serious and is being addressed by many scientists all over the world [23,24,25,26,27]. Research articles about the presence of disinfection by-products (DBPs) in chlorinated water, such as THMs, haloacetic acids, and inorganic chloramines, are being written in various countries [28,29]. There are no unified permissible levels of DBPs in water; every country has its own water quality legislation. In Poland, the basic legal act determining the quality of water in indoor swimming pools is the Regulation of the Minister of Health of 9 November 2015 [30]. Scientists concluded that the major role in DBPs formation is played by water technology management strategies and individual pool characteristics [6,31,32]. The results of investigations showed that outdoor heated pools have higher DBPs levels than unheated ones. Chlorine is used to disinfect the water, it reacts with inorganic and organic matter present in water which results in formation of various by-products. Longer stay in indoor swimming pool halls, from which THMs are not effectively removed, can cause cancer, occupational asthma, rhinitis, upper respiratory tract diseases, and skin and eye irritation [33]. Especially harmful is the effect of THM on children’s organisms in school swimming pools with bad water quality [34,35,36,37]. Therefore, it seems advisable to carry out works aimed to monitor and improve the functioning of the most important technological systems (also related to ensuring appropriate water parameters). PLC controllers are used by scientists in various types of experiments. This is justified by the fact that the highest control standards are fully available, but above all they are reliable and can work in difficult environmental conditions [38]. Therefore, you can easily check the results of laboratory tests in laboratories target locations. Additionally PLC programming environments are equipped with libraries of various types, including regulation algorithms that can be adapted to experiment. Particularly when we are talking about PID regulations [39]. On the other hand, PLCs can be easily integrated with other programming platforms, which allows sharing, processing and archiving data easily [40]. Thanks to advanced libraries, it is also possible to create efficient operator panels, which, similarly to PLC controllers, can work in difficult environmental conditions. Considering harsh environment in technical rooms of swimming pools we chose a control system based on a PLC controller, which will also exchange data with a management system. The main goal of this article is to analyze the kinetics of free chlorine in swimming pool water using constructed analytical device. The results were used to determine the value of reaction rate constant of free chlorine decomposition, which is necessary to prepare a mathematical model of THM formation prediction in swimming pool water. This model is a milestone in a research project “Intelligent HVAC Optimizer with function to detect THM formation potential in indoor swimming pools (OHT)—a new method of electricity and heat management that continuously adjusts ventilation, heating and air conditioning (HVAC) to chlorinated methane by-products concentrations such as trihalomethanes and their removal from indoor swimming pools”. Online water quality control, especially determination of THM formation potential using data received from analytical device, is a novelty in pool technology in comparison to standard procedures which use offline gas chromatography analysis.

## 2. Analytical Device of the Pool Technology

The algorithm presented below is designed to control an analytical device, which is an element of intelligent optimizer (OHT). This project is based on combination of current ventilation management and water treatment process. Such combination only existed in a concept stage, mainly because of the difficulty of real-time THM detection.

The first version of the analytical device [41], which was used to perform the preliminary tests of pool water quality in laboratory conditions and on a real swimming pool facility, was the starting point for the development of the version presented in this article. In the new version of the device, among others, reactor liquid level sensors, a thermocouple, and a heater were added; the type of circulation pump was changed; and the algorithms of the device control were modified.

### 2.1. Elements of the Analytical Device

**Figure 1:**
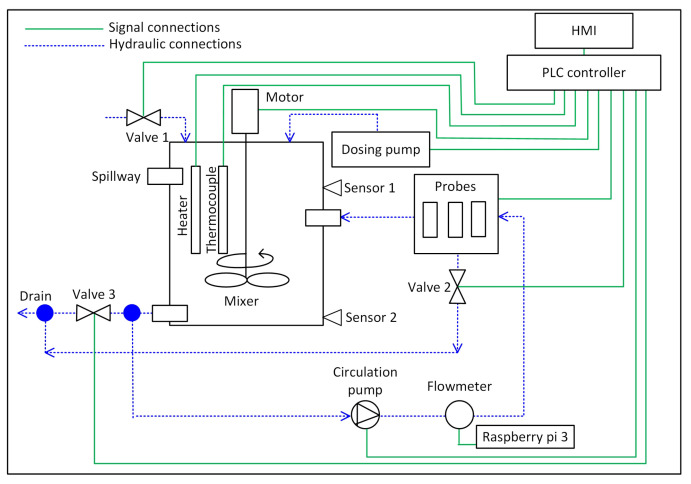
General scheme of the analytical device.

**Figure 2:**
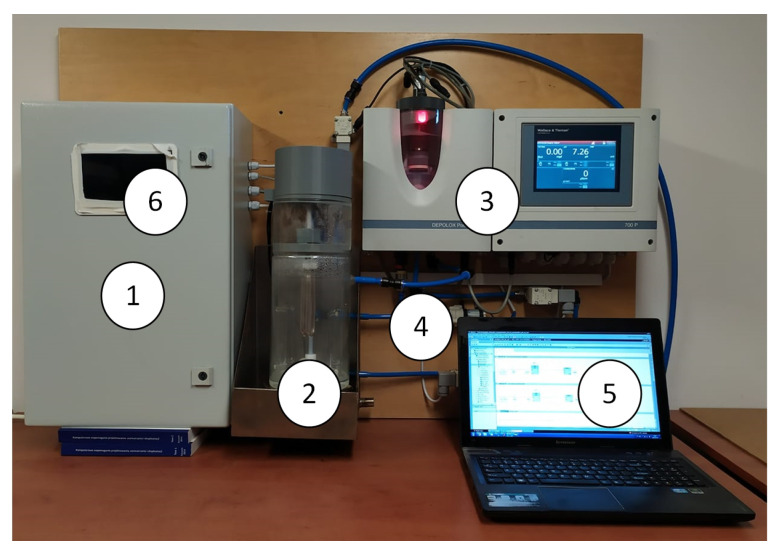
Test stand: 1: control cabinet, 2: reactor, 3: set of probes with the controller, 4: hydraulics system, 5: computer, 6: HMI.

The assumption of authors was to propose a solution which would imitate a small scale pool water circulation. The main elements of this device are (Figure 1 and Figure 2) [42] as follows.

Reactor equipped with a motor: a glass vessel equipped with several devices necessary to ensure optimal conditions for swimming pool water quality testing. It was equipped with a motor, agitator, heater, thermocouple, and liquid level sensors.

Agitator: a mixing device to ensure the homogeneity of the tested liquid. The agitator is coated with polytetrafluoroethylene to ensure sterility of the device. The adjustable rotation speed allows for the selection of the device working parameters.

Heater: a device used to achieve a set temperature of the liquid in the reactor in a specified time and then to maintain a constant temperature with an accuracy of ±0.1 °C during the entire experiment. The reactor uses a 1500 W heater. Set temperature during the experiment was maintained by an automatic control system consisting of a programmable logic controller, thermocouple, and heater.

Valves: valves allow to control the flow of liquid through the analytical device (filling, rinsing, and maintaining the desired water level during the experiment and controlling the flow of water through individual components of the analytical vessel). The filling valve (Valve1) is responsible for filling the reactor with swimming pool water, the probe drain valve (Valve2) allows emptying the probe tank during system rinsing, and the reactor drain valve (Valve3) is used to drain the reactor during the flushing of the device and between the experiments.

Liquid level sensors: the main task of the sensors was to protect devices operating in the reactor and the measuring probes. The upper level sensor (Sensor1) informs about the presence of the required amount of pool water to carry out the experiment, the lower level sensor (Sensor2) informs about the end of the rinsing and emptying process after the experiment is done.

Circulating pump: provides a fixed flow of pool water during the experiment. Ensuring the flow rate with given parameters was necessary due to the requirements of measuring probes chemical parameters of swimming pool water.

Dosing pump: with a capacity of 0.006–190 [cm${}^{3}$× min${}^{-1}$], this device allows for the introduction of sodium hypochlorite into the tested liquid during the experiment at specific doses (calculated by the control system). This allowed us to examine changes in pool water quality and chemical reactions taking place in the pool on a microscale.

Set of measuring probes: membrane probes were used to measure, among others, the concentration of free chlorine, total chlorine, pH, redox potential, temperature, and conductivity, which allowed to carry out tests of pool water quality and the nature of water quality changes during the injection of specific doses of sodium hypochlorite into the reactor.

Thermocouple: allows the measurement of temperature in the reactor. It was included in the automatic control system of the analytical vessel responsible for rapid water heating and maintaining a constant temperature during the test. In order to ensure these parameters in the entire analytical device (reactor and probe tank), the control system uses temperature readings taken in the reactor and probe tank.

Control system: a system that implements an algorithm that controls the analytical device. Its main tasks included control of water flow in the vessel, automatic temperature regulation, and control of water level in the reactor. Such a solution allowed for conducting a cyclic and fully automated process of pool water quality testing without the intervention of an operator. In addition, water temperature control provides a significant reduction in the time needed to conduct the experiment.

Figure 2 shows the test stand.

### 2.2. Sensors and Measurement Probes

Contactless sensors of type XKC-Y25-T12V were used to monitor liquid level in the reactor.

**Figure 3:**
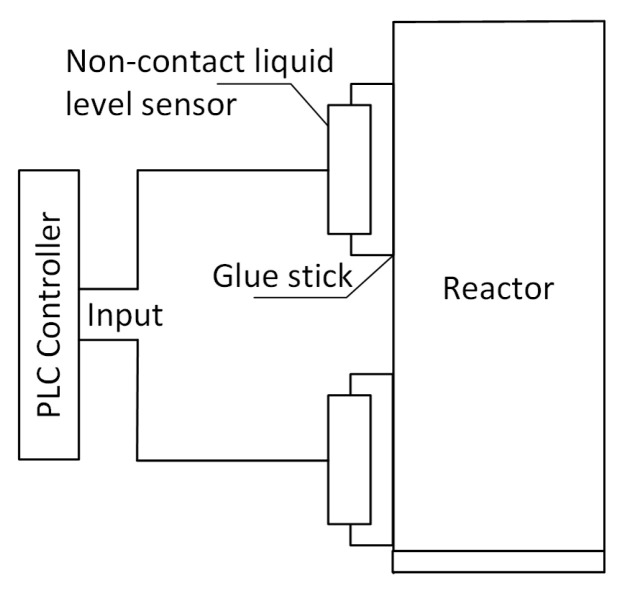
Placement of liquid level sensors.

The sensors were attached to a glass reactor (Figure 3). The main task of the liquid level sensors was to inform the control system about the maximum and minimum liquid level in the reactor. Activation of the upper sensor (Sensor1) is equivalent to the information that the analytical vessel has been filled according to the requirements, i.e., the appropriate amount of water is in the reactor and the probe tank. The lower level sensor (Sensor2) indicates the completion of the rinsing and emptying process after the experiment. This ensured the start of subsequent tests with a new portion of pool water filled into a clean (rinsed) analytical vessel.

**Figure 4:**
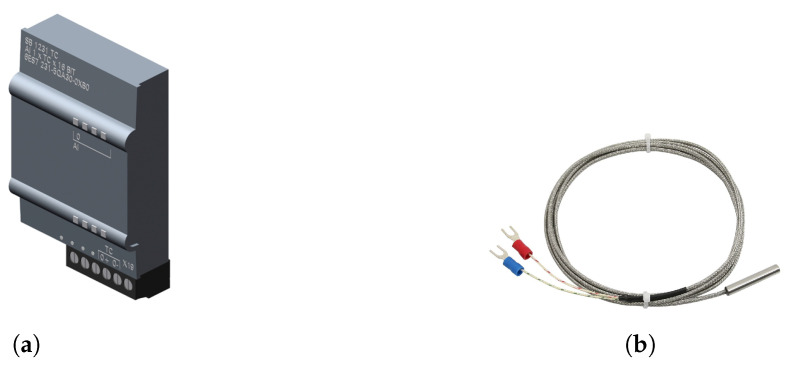
General view of the (**a**) SB1231TC module and (**b**) TJ400 thermocouple.

Cyclic measurement of water temperature in the reactor was performed by means of TJ400 thermocouple, which was connected to the PLC using SB2131TC module (Figure 4). To ensure that the water heats up quickly and the temperature is constant in the entire analytical device (reactor and probe tank), the control system compares the temperature readings from the reactor and probe tank.

**Figure 5:**
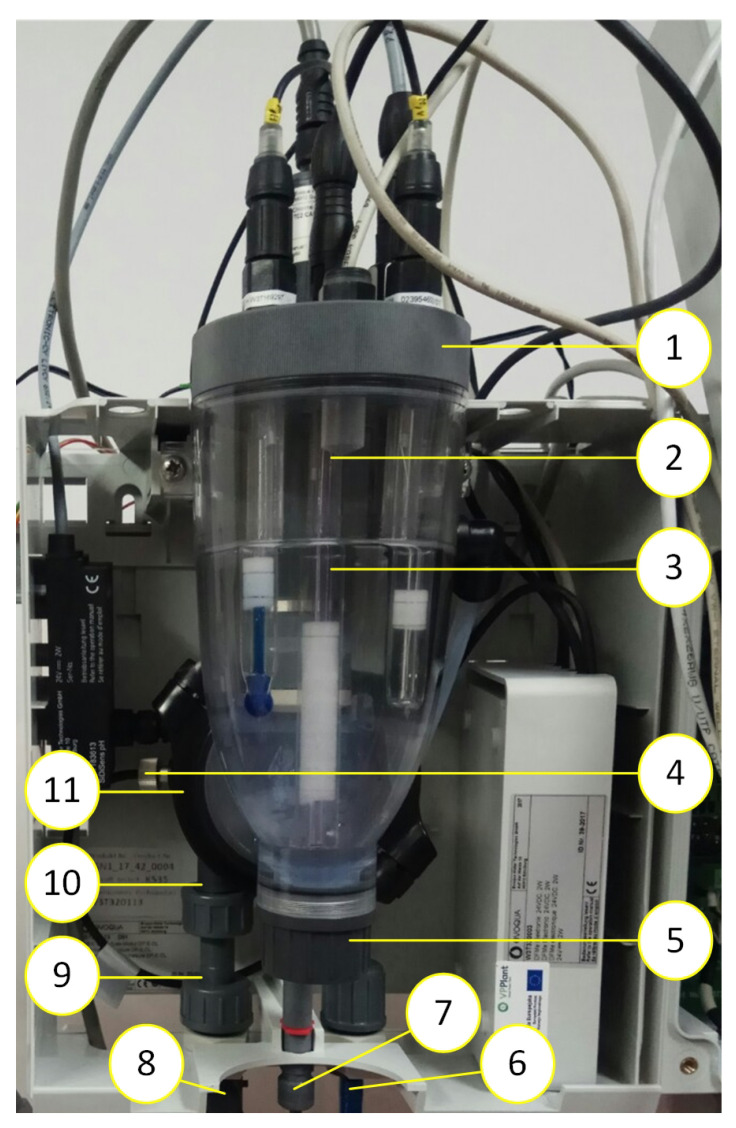
Measuring probes tank list of elements: 1: cover, 2: Housing, 3: Cell body, 4: Multisensor, 5: Flow distribution cap, 6: Sample water outlet, 7: Sample extraction unit, 8: Sample water inlet, 9: Filter unit, 10: Check valve housing, 11: Glow control valve.

To measure water parameters (free chlorine concentration, total chlorine, pH, redox potential, temperature, and conductivity) membrane probes were used (Figure 5), which allowed to carry out quality tests of swimming pool water and testing the nature of water quality changes during the introduction of specific doses of additional substances into the reactor.

The free chlorine sensor is a amperometric 3-electrode system, where the HOCl molecule reacts at the cathode, which gives current proportional to its concentration. The pH sensor is a combined glass electrode, with Ag/AgCl as reference electrode and KCl solution as electrolyte. The oxidation reduction potential (ORP) sensor is a glass shaft with a platinum or gold tip fused into its lower end. It contains an Ag/AgCl electrode as a reference and measures the potential against the metal electrode [43].

#### Control System

The analytical device control system has been made on the basis of SIMATIC S7-1200 driver of SIEMENS Company. The driver enables

- automatic control of the device’s work and monitoring of the control process,
- control of devices (agitator speed and circulating pump capacity) in manual operation, and
- communication with the controller module in order to obtain data on water parameters.

**Figure 6:**
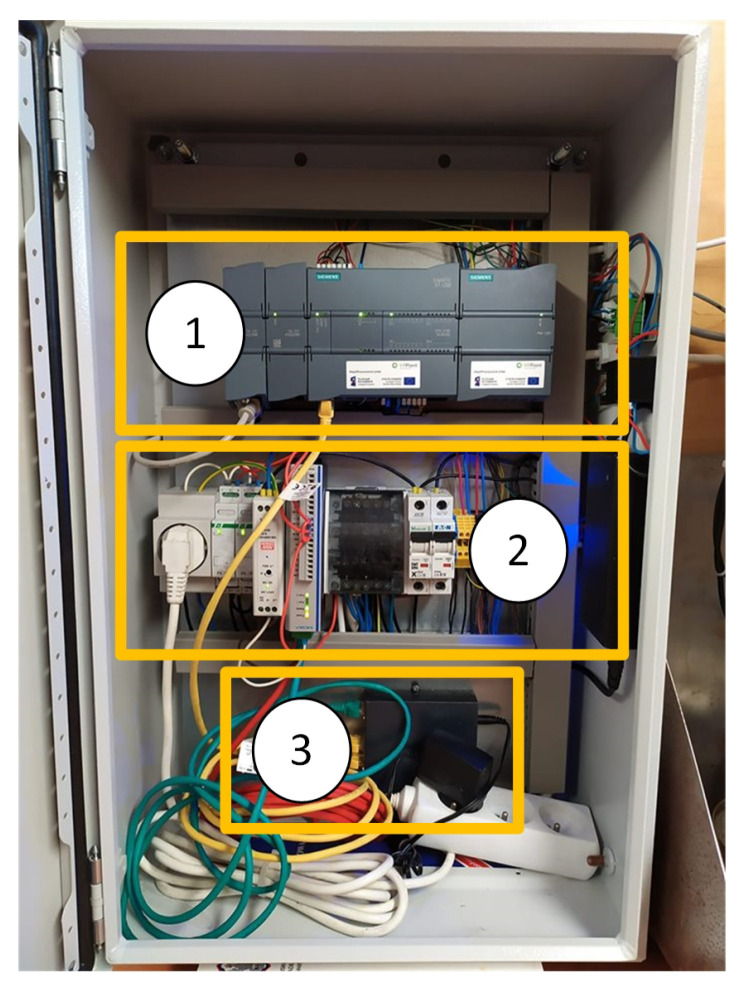
General view of the control cabinet 1: PLC controller, 2: power section, 3: Ethernet switch.

**Figure 7:**
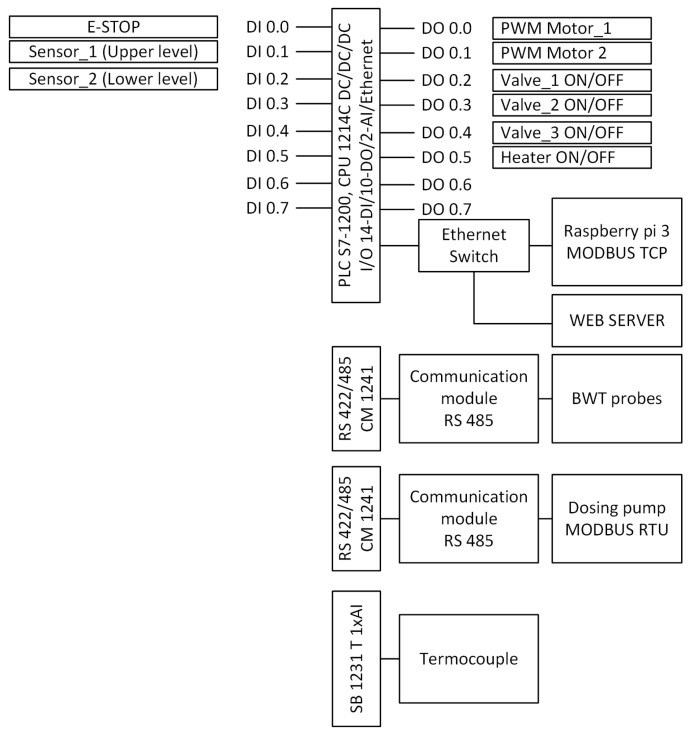
Description of PLC driver inputs/outputs (concept 1).

The control system consists of (Figure 6 and Figure 7)

- PLC driver: the PLC driver (SIMATIC S7-1200, CPU 1214C DC/DC/DC) consists of a central unit, power supply, RS 485 module, analog module (Figure 4a), and Ethernet switch. The driver’s task is to implement a selected operational algorithm of an analytical device [44];
- HMI panel (SIMATIC HMI 7” touch panel): it is connected to driver with the Profinet protocol. The HMI panel is an operator interface; it ensures selection of appropriate operational algorithm, current change of operational parameters, as well as illustration of key functions and states of the analytical device control system; and
- agitator speed and pump capacity monitor: is a system based on Raspberry Pi 3 computer. The task of the Raspberry Pi is to conduct appropriate calculations based on data from sensors (flow meter and speed sensor) and display the agitator speed [Revolutions Per Minute] and circulating pump capacity [mL × min${}^{-1}$].

## 3. Operational Algorithm of An Analytical Device

**Figure 8:**
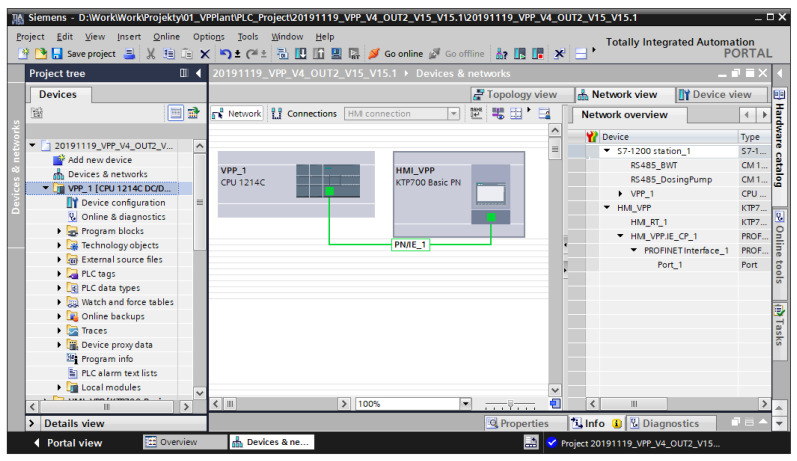
General view of control project in TIA Portal.

The integration of the devices and the control program were developed using the TIA Portal design platform (Figure 8), which allowed for solution testing in the form of computer simulations before the software was implemented on a real workstation [44,45].

**Figure 9:**
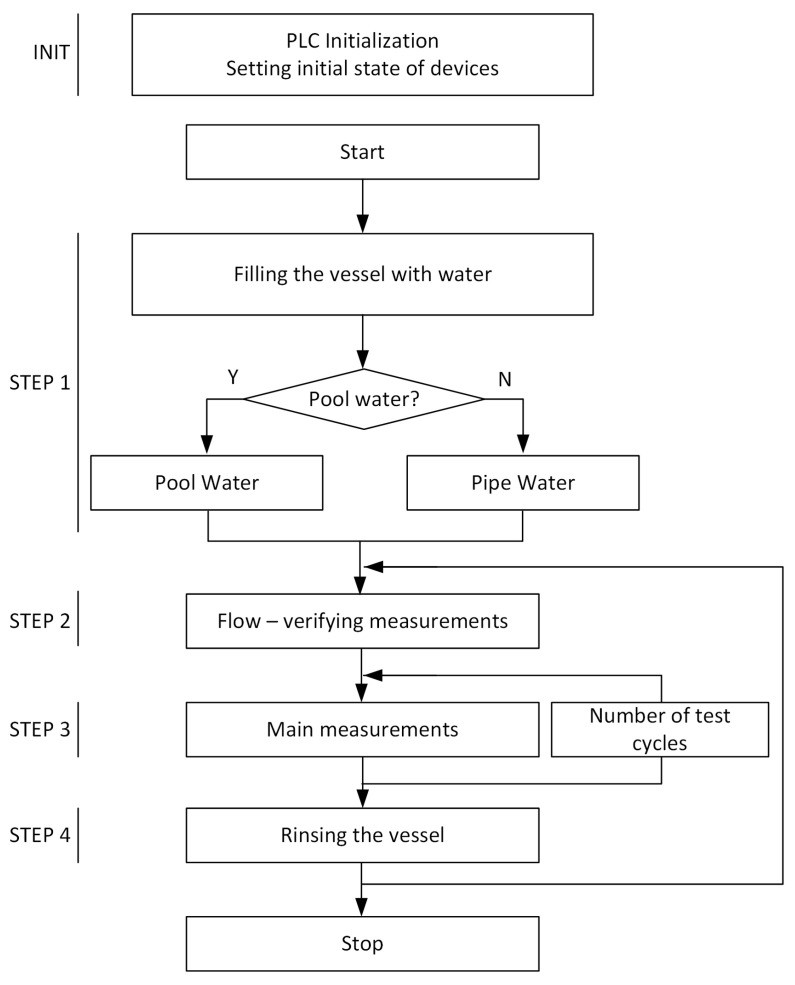
Operational algorithm of the device.

The operational algorithm is shown in Figure 9.

Operational algorithm is divided into five stages:

- Initialization, where the initial values are set (e.g., initial agitator speed and valve states).
- STEP 1: in which the operator decides whether to fill the device with pool water or sewage water, and, after approving the selection, the filling valve is opened until the level determined by the upper level sensor is reached. After filling the device, the reactor and measurement probes are rinsed, for this purpose the agitator and circulation pump are started, and the drain valve is opened.
- STEP 2: in which the analytical device is refilled with water, the circulation pump and the agitator are started, the flow meter is used to check the parameters of liquid flow through the measuring probes, and the obtained results are compared with the reference values.
- STEP 3: in which the main measurement is carried out. In this step, the current water parameters (free chlorine concentration) are compared with assumed parameters and by means of a dosing pump the appropriate chlorine portions are added so that the water parameters are in accordance with expected.
- STEP 4: in which the device is rinsed before the next measurement series begins. For this purpose, the drain valve of the reactor is opened, after complete emptying of the reactor, indicated by a lower level sensor, the drain valve is closed and the station is ready for operation. In this step, the measuring probe tank is not emptied.

Monitoring the process in automatic mode is carried out using the HMI panel on two screens:

- VPP screen
- SEQ screen

**Figure 10:**
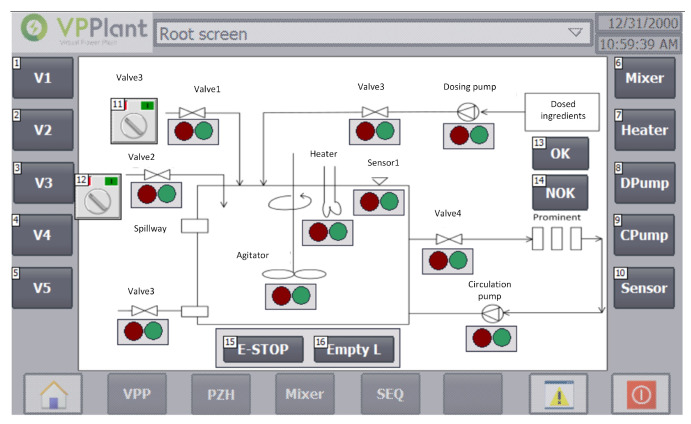
VPP screen—Application control and device status monitoring screen.

The VPP screen (Figure 10) informs on the status of individual devices (switched on: green lamp lights up; switched off: red lamp lights up).

**Figure 11:**
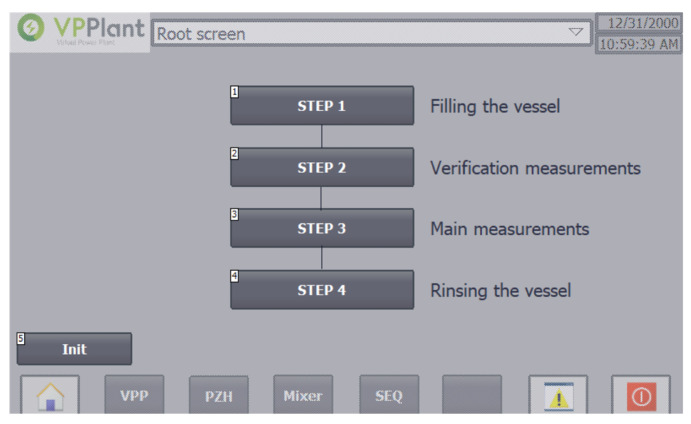
SEQ screen—Control steps monitoring screen.

The SEQ screen (Figure 11) enables to track the process. When operating in automatic mode, the step in which the process is located is highlighted.

## 4. Results

Using the analytical device shown in Figure 2, in the National Institute of Public Health—State Hygiene Facility laboratory tests were carried out, consisting of continuous recording of pool water parameters (free chlorine, total chlorine, pH, redox potential, temperature, and conductivity) in the device during decrease in free chlorine concentration over time. Despite acquiring extensive amount of data, mainly consisting of valuable information about pool water quality, the only information that was significant to determine reaction rate constant was free chlorine concentration changes over time. Therefore, this article shows only part of our data results. Ultimately, the analytical device is to carry out such experiments automatically in the pool to determine the potential for formation of THM in the pool water.

The rate of chemical reaction is defined as the increase in concentration of reaction products (or loss of substrate concentration) in a unit of time. Reaction rate is a parameter that cannot be predicted from the stoichiometric sum reaction equation. One of methods for determining the reaction order is to create a relation of growth or loss of one of the reagents in time. For a simple reaction case A → products can be written by appropriate relation defining its velocity.

(1) $$\nu =\frac{1}{\nu_{A}}\frac{dc_{A}}{dt}=kc_{A}^{\alpha }$$

where

ν: reaction rate (unit depends on reaction order) [e.g., mol × dm${}^{-3}$× s${}^{-1}$],

*νA*: stoichiometric factor of reagent A,

*cA*: instantaneous concentration of reagent A [mol × dm${}^{-3}$],

*t*: time [h],

*k*: rate constant,

α: order in relation to reagent A.

On the basis of the free chlorine decrease measurements, it was found that free chlorine decrease is a reaction of 1st order, i.e., α in Equation (1) is equal to 1, and takes the form described by the relation (2):

(2) $$\nu =\frac{1}{\nu_{A}}\frac{dc_{A}}{dt}=kc_{A}$$

Solved kinetic Equation (2) has a form of

(3) $$\ln c_{A}-\ln c_{0A}=\nu_{A}kt$$

**Figure 12:**
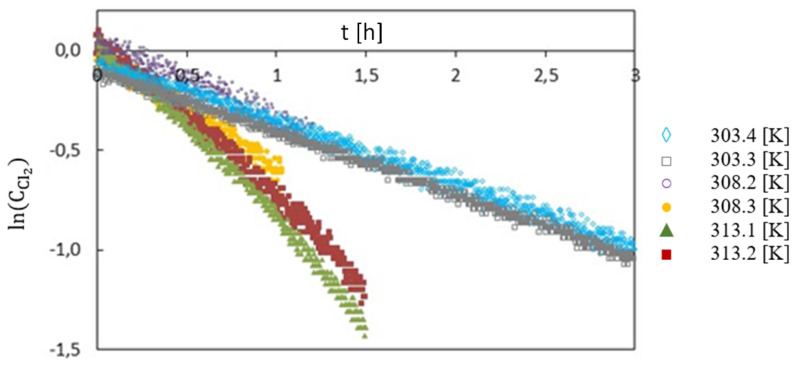
Natural logarithm relation of free chlorine concentration over time for different temperatures.

On the basis of solved kinetic equation of the natural logarithm relation of chlorine concentration as a function of temperature, the values of constant reaction velocity were determined (Table 1; Figure 12).

Quantitative relation of the reaction velocity to temperature was defined by Svante August Arrhenius in the equation:

(4) $$k=Ae^{\frac{Ea}{RT}}$$

where

*k*: constant reaction velocity,

*A*: Arrhenius pre-exponential factor,

*Ea*: activation energy [J× mol${}^{-1}$],

*T*: temperature [K],

*R*: gas constant 8314 [$J\times K^{-1}$× mol${}^{-1}]$.

After logarithmic the equation takes a form of

(5) $$lnk=lnA-\frac{Ea}{RT}$$

**Figure 13:**
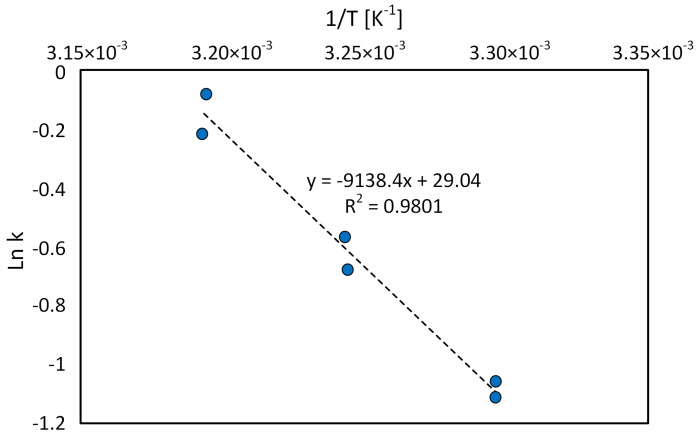
Natural logarithm relation from constant reaction rate of chlorine decay free from inverse temperature.

From Equation (5) it can be concluded that the relation of natural logarithm from the constant reaction velocity to the inverse of temperature is a linear relation (Figure 13).

Based on the directional coefficient of this line, the experimental value of activation energy has been determined: $E_{a}=76.0\;[kJ\times$ mol${}^{-1}]$.

## 5. Discussion

This article presents the concept of controlling the work of an analytical device. On its basis, algorithms and programs controlling the device were prepared. Operational algorithm of PLC driver cooperating with the HMI panel has been prepared for developed device diagram. The whole work cycle was divided into steps for which software modules were developed. The developed control program is scalable and can be easily modified due to its modular structure. The operational process of device is divided into four basic stages: filling device with water, verification measurements, main measurement, and rinsing the device without taking measurements. The program was tested in computer simulation mode and with the use of a real analytical device. The work cycle of the device has been obtained in accordance with assumptions.

One of the important parameters of swimming pool water quality, which describes the presence and quantity of THM in water and air, is the value of constant reaction rate. This value provides information about the kinetics of the reaction. The article presents it in the form of linear change of free chlorine concentration over time.

As shown in results section, a number of experiments were carried out, but only significant data, consisting of free chlorine concentration changes over time, was presented. On the basis of the measurements of free chlorine concentration decrease at a certain temperature (303.4 K), it was found that the decrease of free chlorine concentration is the 1st order reaction (Figure 12; Equation (3)). The analytical device is able to perform within various temperatures of chlorinated water, reaffirming the assumptions made during designing the device. The automatic device control system allows to achieve the set temperature within 2 min and maintain it during the test with the accuracy of ±0.1 K. After a series of consecutive tests at various temperatures, a linear relationship was obtained as a logarithm of chlorine concentration in function of time. The obtained results indicate that a change in water temperature does not cause a change in the reaction rate (Table 1), but it affects the value of the reaction rate constant [46]. Using the Arrhenius equation, we obtained a linear correlation from which experimental value of activation energy was calculated (Figure 13). A low value of activation energy informs about the speed of the reaction (the lower the value, the faster the reaction speed is) [47].

## 6. Conclusions

The lack of sanitary control of water quality in swimming pools increases the risk of infections. The need to monitor THM formation potential is a very significant problem in the case of swimming pool water. Water quality control, on a daily basis, using standard gas chromatography analysis is almost impossible for most of the swimming pools. The article presents an analytical device for online monitoring of the values of reaction rate constant and activation energy. Multiple tests were carried out using the proposed device. The experimental value of activation energy has been determined as $E_{a}=76.0\;[kJ\times$ mol${}^{-1}]$; this value provides information about the reaction speed. Obtained results also indicate that the value of reaction rate constant is depending on the water temperature. Analytical device has been designed to work with HVAC optimizer, in the function of monitoring the potential of creating and removing harmful substances from swimming pools that are by-products of the water disinfection process. By online monitoring of the values of rate constant or activation energy, which is a novelty in swimming pool technology, a new approach to pool water quality assessment can be developed.

## Figures and Tables

**Table 1:** Values of the rate constant as a function of temperature (different dates).

T	$1/T\times 10^{3}$	k	ln k	Date
[K]	$\left[K^{-1}\right]$	[$h^{-1}$]	-	of Experiment
303.4	3.30	0.331	−1.107	21.06
303.3	3.30	0.345	−1.054	9.07
308.2	3.24	0.511	−0.672	5.07
308.3	3.24	0.572	−0.559	21.06
313.1	3.19	0.932	−0.070	4.07
313.2	3.19	0.813	−0.207	6.07
